# Characterization of two functional NKX3.1 binding sites upstream of the *PCAN1 *gene that are involved in the positive regulation of *PCAN1 *gene transcription

**DOI:** 10.1186/1471-2199-9-45

**Published:** 2008-05-04

**Authors:** Wenwen Liu, Pengju Zhang, Weiwen Chen, Chunxiao Yu, Fuai Cui, Feng Kong, Jianye Zhang, Anli Jiang

**Affiliations:** 1 Institute of Biochemistry and Molecular Biology, Shandong University School of Medicine, Jinan, 250012, China

## Abstract

**Background:**

*NKX3.1 *and *PCAN1 *are both prostate-specific genes related to prostate development and prostate cancer. So far, little is known about the regulatory mechanisms of the expression of these two genes. In the present study, we found that NKX3.1 upregulated *PCAN1 *gene transcription in LNCaP prostate cancer cells. To understand the regulatory mechanisms, our work focused on identifying the functional NKX3.1 binding sites upstream of the *PCAN1 *gene, which might be involved in the positive regulation of *PCAN1 *expression by NKX3.1.

**Results:**

We cloned and characterized a 2.6 kb fragment upstream of the *PCAN1 *gene. Analysis of the 2.6 kb sequence with MatInspector 2.2 revealed five potential binding sites of NKX3.1 transcription factor. Luciferase reporter assays, electrophoretic mobility shift assays, chromatin immunoprecipitation and RNA interference were performed to study the effects of NKX3.1 on *PCAN1 *gene expression in prostate cancer cells. Our results showed that *PCAN1 *promoter activity and mRNA expression were increased by transfection with the *NKX3.1 *containing plasmid (pcDNA3.1-*NKX3.1*) and that *PCAN1 *mRNA expression was decreased by RNA interference targeting human *NKX3.1 *in LNCaP prostate cancer cells. The results of electrophoretic mobility shift assays and chromatin immunoprecipitation showed that NKX3.1 bound to NBS1 (-1848 to -1836) and NBS3 (-803 to -791) upstream of the *PCAN1 *gene. The luciferase reporter assays showed that NBS1 and NBS3 enhanced the promoter activity in pGL_3_-promoter vector with cotransfection of the *NKX3.1 *containing plasmid. Furthermore, the deletion of NBS1 or both NBS1 and NBS3 reduced *PCAN1 *promoter activity and abolished the positive regulation of *PCAN1 *expression by NKX3.1.

**Conclusion:**

Our results suggested that two functional NKX3.1 binding sites located at -1848 to -1836 and -803 to -791 upstream of the *PCAN1 *gene were involved in the positive regulation of *PCAN1 *gene transcription by NKX3.1.

## Background

Prostate cancer is the most frequently diagnosed neoplasia in men and one of the leading causes of cancer-related deaths in men over 60 [1]. Although early prostate specific antigen (PSA) detection and surgery have decreased the death rate, most of the patients still die of metastasis and recurrence of prostate cancer [2,3]. However, the mechanisms involved in the onset and progression of prostate cancer are poorly understood at the molecular level. It is important to understand the molecular biology of this cancer for its prevention, early diagnosis, and effective treatment.

*PCAN1 *(prostate cancer gene 1, also known as *GDEP*) is highly expressed in prostate epithelial tissue and frequently mutated in prostate tumors [4-6]. *PCAN1 *expression is undetectable in the highly undifferentiated DU145 and PC-3 prostate cancer cell lines and weakly detected in the more differentiated LNCaP cell line [5]. This gene is localized to chromosome 4q21, a region of the genome that experiences frequent loss of heterozygosity (LOH) in prostate cancer. It is mutated in 35% of the tumor samples [5]. Therefore,*PCAN1 *has been proposed to have tumor suppressing function in prostate cancer.

*NKX3.1 *is a prostate-specific homeobox gene that is thought to play an important role in the normal development of prostate and carcinogenesis. In mice *Nkx3.1 *is exclusively expressed in prostate epithelium [7,8]. Its targeted disruption leads to aberrations in prostate ductal morphogenesis and secretory protein production, and epithelial hyperplasia and dysplasia [9]. Notably *Nkx3.1 *mutant mice display the pathologic changes of prostatic intraepithelial neoplasia (PIN) [10] that is the presumed precursor to prostate cancer in humans, implying that loss of *Nkx3.1 *expression correlates with the initiation of prostate carcinogenesis. Human *NKX3.1 *has been mapped to human chromosome 8p21, a region with frequent loss of heterozygosity in human prostate cancer [11]. This gene has been proposed to have tumor suppressing function [12]. It also inhibits the growth of cultured prostate cancer cells [13-15]* in vitro*. The strong association of *NKX3.1 *with prostate development and prostate cancer makes this gene an attractive molecular target for further study.

*NKX3.1 *and *PCAN1 *are both prostate-specific genes strongly related to prostate development and prostate cancer. Studying the regulatory mechanisms of their expression is important for understanding their roles in prostate development and prostate cancer. In this study, we cloned and characterized a 2.6 kb fragment upstream of the *PCAN1 *gene. Analysis of the 2.6 kb sequence with MatInspector 2.2 revealed potential binding sites of some important transcription factors, including NKX3.1, P53, Sp1, cEBP and PPAR/RXR heterodimers. In our study, the eukaryotic expression plasmids containing *NKX3.1, p53, Sp1, cEBPα *and *PPARγ *were respectively used to study their effects on *PCAN1 *expression. We found that NKX3.1 upregulated *PCAN1 *promoter activity and mRNA expression. To explore the regulatory mechanisms of NKX3.1 on *PCAN1 *transcription, we focused on identifying the functional NKX3.1 binding sites (NBSs) upstream of the *PCAN1 *gene. We demonstrated that NKX3.1 upregulated *PCAN1 *gene transcription through direct binding with NBSs upstream of the *PCAN1 *gene. Our study provided a molecular mechanism for the regulation of *PCAN1 *gene expression.

## Results

### Effects of NKX3.1 on *PCAN1 *promoter activity and mRNA expression

**Figure 1:**
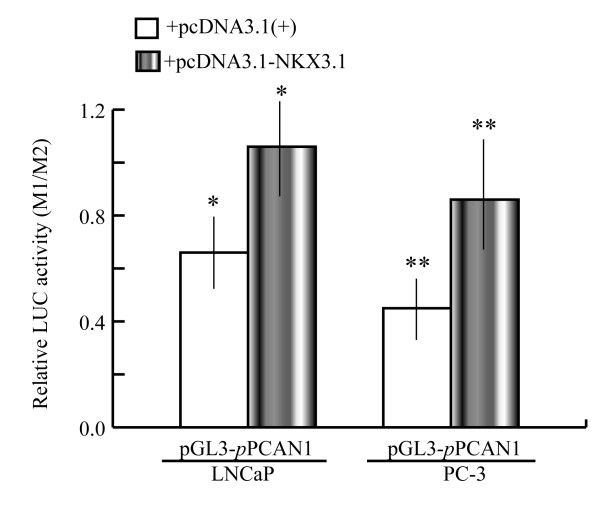
**Effects of NKX3.1 on the *PCAN1 *promoter in LNCaP and PC-3 cells**. LNCaP and PC-3 cells were cotransfected with pGL_3_-*p*PCAN1 and pcDNA3.1-*NKX3.1 *(grey bars). The control cells were cotransfected with pGL_3_-*p*PCAN1 and pcDNA3.1 (+) plasmid (blank bars). The cells were harvested 48 h after transfection and *PCAN1 *promoter activity was detected by dual-luciferase reporter assays. Results were expressed as relative luciferase activities (M1/M2). The data were represented as the mean of four individual values ± SD.*p < 0.05, **p < 0.05, grey bar *vs *blank bar.

In our previous work, a 2.6 kb promoter fragment (+32 to -2598) of the *PCAN1 *gene amplified by PCR was inserted into pGL_3_-basic vector to form the *PCAN1 *promoter-luciferase reporter plasmid designated as pGL_3_-*p*PCAN1. Firefly luciferase expression driven by the 2.6 kb *PCAN1 *promoter was used to evaluate the promoter activity [16]. To detect the effects of NKX3.1 on *PCAN1 *gene expression, LNCaP and PC-3 cells were harvested 48 h after cotransfection with pGL_3_-*p*PCAN1 and pcDNA3.1-*NKX3.1*. The control cells were cotransfected with pGL_3_-*p*PCAN1 and pcDNA3.1 (+) plasmid. The *PCAN1 *promoter activity detected by dual-luciferase reporter assays was enhanced by 1.6-fold in LNCaP cells and by 1.8-fold in PC-3 cells with *NKX3.1 *cotransfection, compared with *PCAN1 *promoter activity in the control cells (Fig. 1).

**Figure 2:**
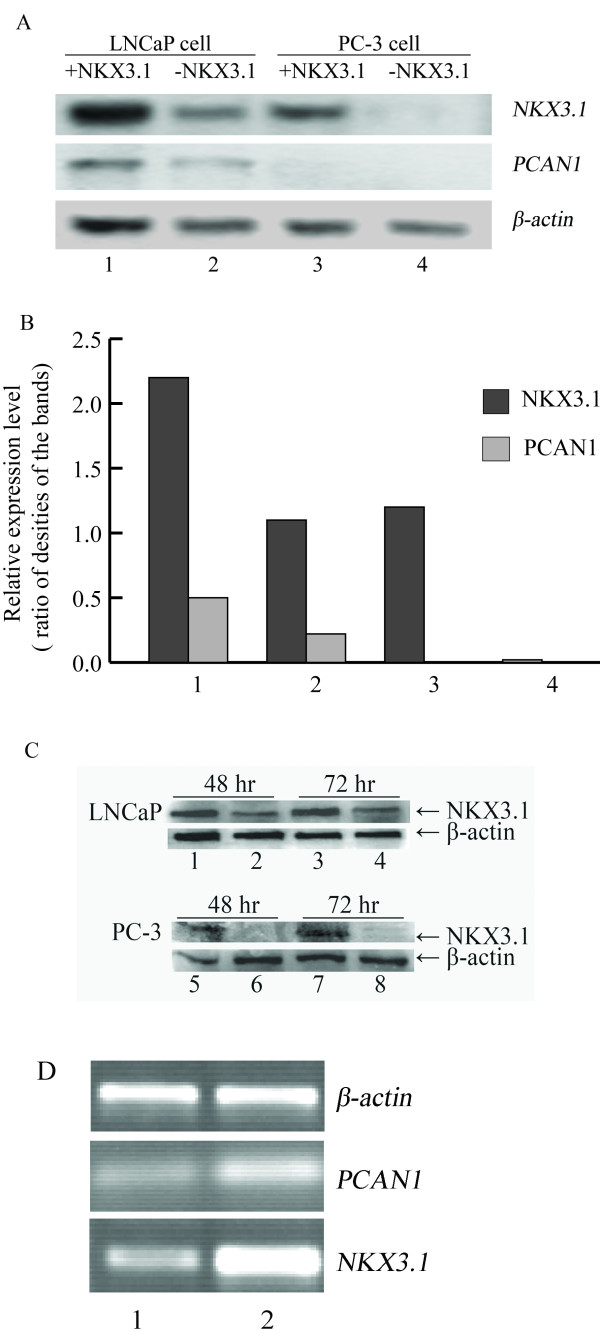
**Effects of NKX3.1 on *PCAN1 *mRNA expression in LNCaP and PC-3 cells**. The effects of NKX3.1 on *PCAN1 *mRNA expression as detected by RT-PCR. **A. ***PCAN1 *mRNA expression following transfection of LNCaP cells with 1. pcDNA3.1-*NKX3.1*; 2. pcDNA3.1 (+); or following transfection of PC-3 cells with 3. pcDNA3.1-*NKX3.1*; or 4. pcDNA3.1 (+). **B. **Relative expression levels were presented as the ratio of densities of *NKX3.1 *or *PCAN1 *to *β-actin *bands. The results were expressed as mean ± SD (n = 3). **C. **The expression of NKX3.1 protein in LNCaP and PC-3 cells after transfection of pcDNA3.1-*NKX3.1 *(1, 3, 5, 7) or pcDNA3.1 (+) (2, 4, 6, 8).**D. ***NKX3.1 *and *PCAN1 *mRNA expression following stable transfection of LNCaP cells with 1. pRNAT-RNAi1 targeting human *NKX3.1*; 2. pRNAT-RiN as a control vector.

The *PCAN1 *mRNA expression level in LNCaP cells, as detected by RT-PCR, was increased significantly by *NKX3.1 *cotransfection, compared with that of the control cells transfected with pcDNA3.1 (+) plasmid (Fig. 2A and 2B). The *PCAN1 *mRNA expression level in PC-3 cells was undetectable by RT-PCR (Fig. 2A and 2B).

The expression of endogenous *NKX3.1 *in LNCaP cells was knocked down by RNAi, which made the *PCAN1 *mRNA expression decrease, as detected by RT-PCR (Fig. 2D).

### Identification of specific NKX3.1 binding sites (NBSs) with nuclear extracts

**Figure 3:**
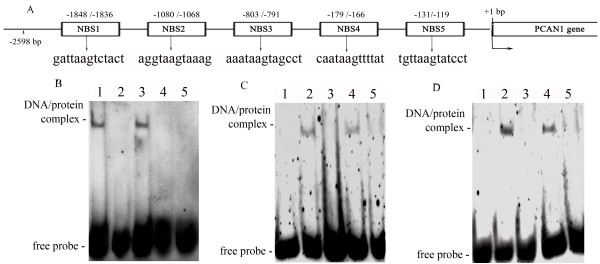
**Binding assays of NKX3.1 binding sites with nuclear extracts**. **A. **Locations and sequences of five potential NKX3.1 binding sites (NBSs) upstream of the *PCAN1 *gene. **B. **EMSA was performed to assay the binding activities of the five NBSs with the nuclear extracts from LNCaP cells. 1–5: labelled NBS1~NBS5 with LNCaP nuclear extracts respectively. A DNA-protein binding complex was formed when the labelled probe of NBS1 (lane 1) or NBS3 (lane 3) was reacted with LNCaP nuclear extracts. **C **and** D. **Competition binding and antibody blocking were used to detect the specific binding of the NBS1 (C) or NBS3 (D) with NKX3.1. Labelled NBS1 or NBS3 without nuclear extracts was used as the control (lane1); a specific DNA-protein complex was formed when labelled NBS1 or NBS3 was reacted with LNCaP nuclear extracts (lane 2). Competition binding was performed in the presence of a 250-fold molar excess of unlabelled NBS1 or NBS3 (lane 3), unlabelled mutant NBS1 or NBS3 (lane 4). The binding activity of probe NBS1 or NBS3 to nuclear extracts could be blocked by anti-NKX3.1 antibody (lane5).

Analysis of the 2.6 kb promoter sequence with MatInspector 2.2 revealed five potential NKX3.1 transcription factor binding sites (sequences are shown in Fig. 3A). They were located at -1848 to -1836 (NBS1), -1080 to 1068 (NBS2), -803 to -791 (NBS3), -179 to 166 (NBS4) and -131 to -119 (NBS5), upstream of the *PCAN1 *gene. To investigate the binding activities of these five NBSs with NKX3.1 transcription factor, we carried out electrophoretic mobility shift assays (EMSA). It was performed with *NKX3.1*-transfected nuclear extracts from LNCaP cells and synthesized oligonucleotide probes containing these five NBS sequences. The results showed that DNA-protein binding complexes were identified for the NBS1 probe and the NBS3 probe (Fig 3B). The bindings of NBS1 and NBS3 with nuclear extracts proved to be specific, as they were blocked by a 250-fold excess of unlabeled NBS1 probe (Fig. 3C) or NBS3 probe (Fig. 3D) and by anti-NKX3.1 antibody (Fig. 3C and 3D), but not by unlabeled mutant NBS1 probe or mutant NBS3 probe (Fig. 3C and 3D).

### NKX3.1 binds to NBS1 and NBS3 upstream of the *PCAN1 *gene in living cells

**Figure 4:**
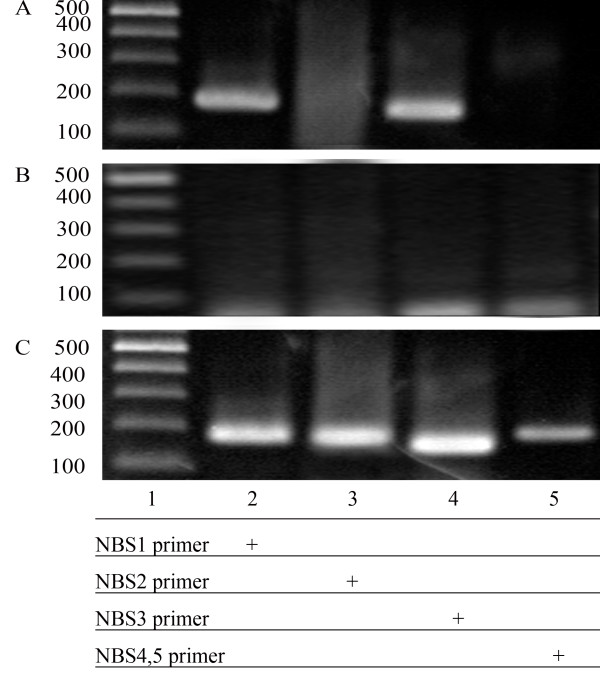
**NKX3.1 binds to the NBSs of the *PCAN1 *promoter in living cells**. LNCaP cells transfected with pcDNA3.1-*NKX3.1 *were cross-linked by formaldehyde treatment and lysed. Cell lysates were subjected to immunoprecipitation with either an antibody to NKX3.1 (A) or rabbit IgG (B). Four primers (names and sequences are shown in Table 1) spanning five NBSs of *PCAN1 *promoter region were used for PCR of recovered DNA from the immunoprecipitation (lanes 2–5). Input DNA was used as positive control (C).

To determine whether NKX3.1 also binds to the NBSs *in vivo*, we performed chromatin immunoprecipitation (ChIP) assays, which are used to define interactions of proteins with specific DNA elements in living cells. ChIP was carried out in LNCaP cells transfected with pcDNA3.1-*NKX3.1*. After cross-linking with formaldehyde, cell lysates were immunoprecipitated with anti-NKX3.1 antibody or rabbit IgG (negative control). The DNA purified from this coprecipitation was analyzed by PCR with primers (sequences are shown in Table 1) spanning the NBSs in the *PCAN1 *promoter. As shown in Fig. 4, we observed a clear PCR product using NBS1 primer or NBS3 primer but no band was observed using NBS2 primer or NBS4,5 primer, and no PCR product was identified for all primers with rabbit IgG precipitation complexes, indicating that NKX3.1 bound to NBS1 and NBS3 upstream of the *PCAN1 *gene in living cells.

**Table 1:** PCR primers used in chromatin immunoprecipitation

Names	Sequences	Product sizes
NBS1 primers	F: GATTCTTTGACTGGTCTGGCACAC	
	R: TTATCCATTGTGCCTGGAGCTGAG	170 bp (spanning NBS1)
BNS2 primers	F: TCCTACTAACGGCATGTAAGGAGG	
	R: ATTGCCATGTCTGGACTGTGAGTG	170 bp (spanning NBS2)
NBS3 primers	F: AAGAATGAGCTGATCCTCCTATGC	
	R: GGTTTAGTAATAGACTGGGCACCA	150 bp (spanning NBS3)
NBS4,5 primers	F: GTGTAGCAGGTAAATCAGTGTGAG	
	R: TCAGCTGACGAGCAACTTCAATTC	180 bp (spanning NBS4 and 5)

NBS1 F and R span NBS1;

NBS2 F and R span NBS2; NBS3 F and R span NBS3; BNS4,5 F and R span NBS4 and NBS5

### Interaction of NKX3.1 with NBS1 or NBS3 stimulates luciferase reporter expression driven by SV40 promoter

**Figure 5:**
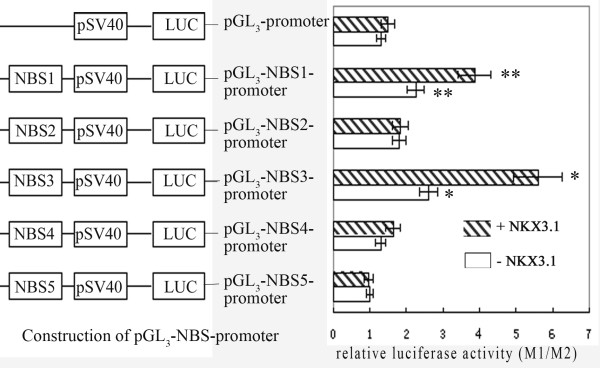
**Effects of NBSs on the SV40 heterogeneous promoter**. The sequences of five NBSs were synthesized *in vitro *and inserted upstream of the SV40 promoter in pGL_3_-promoter vector generating the pGL_3_-NBS-promoter plasmids. The five recombinant pGL_3_-NBS-promoter plasmids or pGL_3_-promoter vector were then cotransfected with pcDNA3.1-*NKX3.1 *respectively, to study the effects of NBSs on the activity of the SV40 heterogeneous promoter. Cotransfection of pGL_3_-NBS-promoter with pcDNA3.1 (+) vector was used as the control. The promoter activities were determined by dual luciferase assays. Results were expressed as relative luciferase activity (M1/M2). The data were represented as the mean of four individual values ± SD. *p < 0.01,**p < 0.01, strip bar *vs *blank bar.

To investigate the interactions between NKX3.1 and these five potential NBSs *in vivo*, five pGL_3_-NBS-promoter luciferase plasmids were constructed (Fig. 5) and cotransfected with pcDNA3.1-*NKX3.1 *plasmid respectively into LNCaP cells, while the control cells were cotransfected with pcDNA3.1 (+) vector. The luciferase reporter assays showed that when cotransfected with *NKX3.1 *expression plasmid, NBS1 and NBS3 enhanced SV40 promoter activity by 1.7-fold and 2.1-fold, respectively, compared with that of the control cells that were cotransfected with pcDNA3.1 (+) vector. NBS2, NBS4, NBS5 showed no significant effects on SV40 promoter activity. These results suggested that NBS1 and NBS3 were the functional *cis*-elements *in vivo *for the upregulation by NKX3.1.

### Deletion of NBS1 and NBS3 in the *PCAN1 *promoter abolishes the positive regulation by NKX3.1

**Figure 6:**
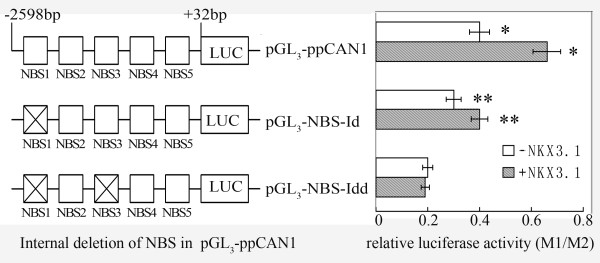
**Effects of deletion of NBS1 and NBS3 on *PCAN1 *promoter activity**. pGL_3_-*p*PCAN1 is a luciferase reporter plasmid containing a 2.6 kb wild type *PCAN1 *promoter; pGL_3_-NBS-Id represented pGL_3_-NBS1id-*p*PCAN1 with deletion of NBS1 from the *PCAN1 *promoter; pGL_3_-NBS-Idd represented pGL_3_-NBS1,3idd-*p*PCAN1, in which both NBS1 and NBS3 were deleted from the *PCAN1 *promoter. They were cotransfected with pcDNA3.1-*NKX3.1 *(grey bar) or pcDNA3.1 (+) vector (blank bar) into LNCaP cells, and the promoter activity was analyzed by dual-luciferase reporter assays. Results were expressed as relative luciferase activities (M1/M2). The data were represented as the mean of four individual values ± SD. *p < 0.01, **p < 0.05, grey bar *vs *blank bar.

The binding assays of five NBSs *in vivo *and *in vitro *suggested that NBS1 and NBS3 in the *PCAN1 *promoter were involved in the positive regulation of *PCAN1 *expression by NKX3.1. To further confirm this observation, the sequences of NBS1, or both NBS1 and NBS3 were deleted from pGL_3_-*p*PCAN1 to examine the effects of the deletions on *PCAN1 *promoter activity. The results in Fig. 6 showed that deletion of NBS1 (pGL_3_-NBS1id-*p*PCAN1) or both NBS1 and NBS3 (pGL_3_-NBS1,3idd-*p*PCAN1) reduced the promoter activity to 75% or 50% of the wild-type promoter (pGL_3_-*p*PCAN1). With cotransfection of the *NKX3.1 *expression plasmid, deletion of NBS1 partially abolished, and deletion of both NBS1 and NBS3 completely abolished *NKX3.1 *stimulation of the *PCAN1 *promoter. These findings suggested that NBS1 and NBS3 upstream of the *PCAN1 *gene were functional *cis*-elements mediating the positive regulation by NKX3.1 of *PCAN1 *gene transcription.

## Discussion

*NKX3.1 *is a prostate-specific homeobox gene that is thought to play important roles in normal prostate development. NKX3.1 protein has been proposed to act as a specific tumor suppressor in prostate. Loss of *NKX3.1 *expression correlates with prostate carcinogenesis [15] and prostate tumor progression [17]. It was reported that NKX3.1 could collaborate with other transcription factors, such as serum response factor (SRF) [18], Sp-family protein [19] and prostate derived Ets factor (PDEF) [20], to regulate the expression of target genes. The potential for NKX3.1 to exert a differentiating and growth suppressing effect on prostatic epithelium was confirmed by targeted gene disruption of *Nkx3.1 *in mice [21]. Deletion of either one or both copies of *Nkx3.1 *resulted in prostatic epithelial hyperplasia and dysplasia that increased in severity with age. Magee et al. [21] has analyzed the expression profiles of prostate tissues from wild-type,*Nkx3.1*^+/-^, and *Nkx3.1*^-/- ^mice and identified Nkx3.1 target genes. However, the genes directly regulated by human NKX3.1 have yet to be identified. In the present study, we found that *NKX3.1 *upregulated the expression of the *PCAN1 *gene in LNCaP prostate cancer cells and identified two functional NKX3.1 binding sites upstream of the *PCAN1 *gene.

*PCAN1 *is highly expressed in prostate epithelial tissue and was initially identified in a screen for prostate-specific genes. As an important gene in prostate cancer initiation or progression [22], it has been shown that *PCAN1 *is frequently mutated or deleted in prostate tumor samples [5] and differentially expressed in tumor versus normal prostate tissue, demonstrating a prostate tumor suppressor role [6]. So far, little is known about the regulatory mechanisms of *PCAN1 *gene expression as well as the relevant regulatory elements and factors. Cross et al. [4] has identified *PCAN1 *gene in prostate epithelial tissue and demonstrated its expression pattern in different cells. They did an initial characterization of 2.5 kb sequence upstream of the initiation sites with the MatInspector program, in which they found several important transcription factor binding sites, including NKX3.1 binding sites. In our study, we cloned a 2.6 kb *PCAN1 *promoter sequence and five NKX3.1 binding sites were found in this region with the MatInspector (core/matrix sim: 0.75/optimized). We further performed reporter assays, RNAi, EMSA and ChIP to demonstrate that NKX3.1 could directly bind to NKX3.1 binding sites in the *PCAN1 *promoter to enhance *PCAN1 *gene expression in prostate cancer cells. This finding provides a foundation for future studies of the regulatory mechanisms and roles of *NKX3.1 *on *PCAN1 *gene expression in prostate development and prostate cancer.

NKX3.1 is proposed to be a nuclear transcriptional factor. It preferentially binds the TAAGTA sequence [23] to regulate its target gene expression. It is reported that NKX3.1 acts as a transcription factor through recruitment of some corepressors [24,25] or coactivators [26,27] to repress or activate its target genes. Our study provided some evidences to show that NKX3.1 directly binds to NKX3.1 binding sites in the *PCAN1 *promoter to activate *PCAN1 *gene expression in prostate cancer cells. However, further studies will be required to explore the regulatory mechanisms and the cofactors involved in the regulation. *NKX3.1 *is a prostate-specific gene required for maintenance of the normal differentiated state of the prostate epithelium. It is proposed to have tumor suppressing function. However, it is not a classic tumor suppressor gene. Despite that loss of function of *NKX3.1 *predisposes to prostate cancer, it is not sufficient for tumorigenesis. Moreover, while one allele of *NKX3.1 *is lost by means of chromosomal deletion in prostate cancer, the other allele does not undergo mutational inactivation. These features are not consistent with activities of "classic" tumor suppressor genes. Instead, *NKX3.1 *appears to act more like a tumor modulator, serving as a regulator of differentiation, which in turn prevents cancer initiation. Our finding that NKX3.1 upregulates *PCAN1 *expression will give a clue for further exploring the relationships between *NKX3.1 *and *PCAN1 *and understanding the functional importance of NXK3.1 in regulating *PCAN1 *in prostate differentiation and carcinogenesis. Identification of the specific biological functions of NKX3.1 in prostate cancer may provide targets for the early diagnosis and prevention of prostate cancer.

*NKX3.1 *is an androgen regulated gene and its expression is upregulated by androgens. It is presumed that androgens upregulate *PCAN1 *expression through increasing *NKX3.1 *expression. In the experiments we have analyzed the effect of androgen (R1881) on endogenous *PCAN1 *and *NKX3.1 *expression in LNCaP cells. Our results showed that R1881 (10^-8^~10^-10 ^M) increased *NKX3.1 *expression but have no significant effects on *PCAN1 *expression in RT-PCR (results are not shown in this paper) and *PCAN1 *promoter activity in luciferase reporter assays (results are shown in our paper in Cell Mol Biol Lett). So far, it is not very clear whether or not *PCAN1 *gene expression is regulated directly by androgens. It may be possible that *PCAN1 *is negatively regulated by androgens and *NKX3.1 *compensates this effect through positively regulating *PCAN1*. In addition, *AR*, *NKX3.1 *and *PCAN1 *play different roles in prostate development and cancer, and their interactions in prostate are very complicated and need further to be investigated.

## Conclusion

In conclusion, we cloned and characterized the human *PCAN1 *promoter, and identified two functional NKX3.1 binding sites upstream of the *PCAN1 *gene, which were involved in the positive regulation by NKX3.1 of *PCAN1 *gene expression. Both *NKX3.1 *and *PCAN1 *are related to prostate development and prostate cancer. Our findings will contribute to the understanding of molecular regulatory mechanisms of *PCAN1 *gene expression in prostate development and cancer.

## Methods

### Construction of the *NKX3.1 *eukaryotic expression plasmid (pcDNA3.1-*NKX3.1*)

pCR2.1-*NKX3.1*, a T-clone of *NKX3.1 *cDNA containing the complete sequence of *NKX3.1 *(a gift from Dr. Charles Young, Mayo Clinics, USA), was digested with *EcoR *I (TakaRa, Shiga, Japan) to release the 971 bp fragment of *NKX3.1 *cDNA. The *NKX3.1 *cDNA sequence (971 bp) contains the CDS of NKX3.1 including a start codon, a stop codon and a partial 3' UTR. It was then inserted into pcDNA3.1 (+) vector (Invitrogen Life Technologies, San Diego, CA, USA), which had been digested with *EcoR *I and dephosphorylated with calf intestine alkaline phosphatase (TaKaRa), to generate the *NKX3.1*-cDNA eukaryotic expression plasmid pcDNA3.1-*NKX3.1*. The recombinant plasmid was digested with *EcoR *I to identify the size of the insert and digested with *Not *I (TaKaRa) to identify the correct insert orientation. The *NKX3.1 *cDNA was also confirmed by DNA sequencing.

### Construction of the *PCAN1 *promoter-luciferase reporter plasmid (pGL3-*p*PCAN1)

The 2.6 kb promoter fragment (+32 to -2598) of the *PCAN1 *gene was amplified by PCR using human genomic DNA as the template. The primer pairs were *PCAN*F 5'-CCCTAGCTAGCCATCTCTGCAGTCTCGAC-3' with a *Nhe *I site at the 5'-end and *PCAN*R 5'-CCCAAGCTTCGCTCTGACTTCCTCTTC-3' with a *Hind *III site at the 5'-end. The PCR was conducted at 96°C for 2 min followed by 35 cycles at 98°C for 20 s, 68°C for 10 min. The amplified fragment was isolated and purified following agarose electrophoresis using a Gel Extract Kit (Omega Bil-Tek. Inc. Doraville, GA, USA), digested with *Nhe *I and *Hind *III (TaKaRa), and ligated into the equivalent sites of the pGL_3_-basic vector (Promega, Madison, WI, USA) to generate the pGL_3_-*p*PCAN1 construct. The resulting construct was confirmed by restriction enzyme digestion and DNA sequence analysis.

### Construction of luciferase reporter plasmids of NKX3.1 binding sites (pGL3-NBS-promoter)

Analysis of the 2.6 kb promoter sequence using MatInspector 2.2 revealed five potential binding sites for the NKX3.1 transcription factor. To confirm the functional binding sites, we synthesized oligonucleotides corresponding to these five sequences (NBS1~NBS5) shown in Table 2. Each NBS sequence was synthesized with an overhanging *Mlu *I site (CGCGT) at the 5'-end of the sense strand and an overhanging *Xho *I site (TCGAG) at the 5'*-*end of the antisense strand. The double-stranded NBS was generated by annealing equal amounts of sense and antisense oligonucleotides at 95°C for 10 min, then cooling to room temperature. The double-stranded NBS was inserted upstream of the SV40 promoter in the pGL_3_-promoter (Promega) vector to generate the recombinant plasmid of NBS-SV40 promoter-luciferase reporter gene. All constructs were confirmed by DNA sequencing.

**Table 2:** Probes used in electrophoretic mobility shift assays

Names	Sequences (sense strand)
NBS1	GATTAAGTCTACT
NBS2	AGGTAAGTAAAG
NBS3	AAATAAGTAGCCT
NBS4	CAATAAGTTTTAT
NBS5	TGTTAAGTATCCT
Mutant NBS1	GATTCCTGCTACT
Mutant NBS3	AAATCCTGAGCCT

### Construction of the NBS deletion plasmids of pGL3-pPCAN1

The construction of NBS1 or both NBS1 and NBS3 deletion plasmids was made by a two-step PCR procedure. In the first step, pGL_3_-*p*PCAN1 was used as the template for the construction of pGL_3_-NBS1id-*p*PCAN1, and two PCR fragments were generated with two primer pairs, NBS1id-up 5'-CCCTGACTTGGGGGGCTCC ACTGTAGTAC-3' and *PCAN*F 5'-CCCTAGCTAGCCATCTCTGCAGTCTCGAC-3', NBS1id-down 5'-TGGAGCCCCCCAAGTCAGGGGGTTTAATC-3' and *PCAN*R 5'-CCCAAGCTTCGCTCTGACTTCCTCTTC-3'. The PCR conditions were 94°C for 2 min, 98°C for 10 s, 68°C for 5 min, 32 cycles. The two PCR fragments were purified by agarose electrophoresis and used together as templates in the second step of PCR with primers *PCAN*F and *PCAN*R. The PCR conditions were 94°C for 2 min, 60°C for 30 s, 72°C for 5 min, followed by addition of *PCAN*F and *PCAN*R primers and PCR at 98°C for 10 s, 68°C for 6 min, 32 cycles. The PCR fragments and pGL_3_-basic were both digested with *Hind *III and *Nhe *I and ligated together to construct pGL_3_-NBS1id-*p*PCAN1. A similar approach was used to generate pGL_3_-NBS1, 3idd-*p*PCAN1, in which both NBS1 and NBS3 sequences were deleted. pGL_3_-NBS1id-*p*PCAN1 was used as the template and other two primer pairs, NBS3id-up 5'-GAATGCCTGGTATTTCTTATCAAGAGAAC-3' and *PCAN*F, NBS3id-down 5'-ATAAGAAATACCAGGCATTCCCTTCCACCA-3' and *PCAN*R, were used. The constructed deletion plasmids were validated by DNA sequencing.

### Cell culture

The human prostate cancer cell lines LNCaP and PC-3 were obtained from the American Type Culture Collection (ATCC). They were grown at 37°C in 5% CO_2 _with RPMI 1640 media (Sigma, St. Louis, MO, USA) supplemented with 10% fetal bovine serum (Gibco, BRL Grand Island, NK, USA), ampicillin 100 U/ml and streptomycin 100 U/ml.

### Transient transfection

For the promoter activity assay of pGL_3_-*p*PCAN1, LNCaP and PC-3 cells were transfected with lipofectamin™ 2000 (Invitrogen) in 24-well plates. Each well included 1.5 × 10^5 ^cells, 1.0 μg pGL_3_-*p*PCAN1, 0.04 μg internal control vector pRL-TK (Promega), 2 μl lipofectamin™ 2000 and 500 μl RPMI 1640 media without serum and antibiotics. The cells were analyzed using a dual-luciferase reporter assay system (Promega) 48 h after completion of the transfection procedure.

For cotransfection experiments of the *NKX3.1 *expression plasmid (pcDNA3.1-*NKX3.1*) with pGL_3_-construct (pGL_3_-*p*PCAN1, pGL_3_-NBS-promoter, pGL_3_-NBS1id-*p*PCAN1 or pGL_3_-NBS1,3idd-*p*PCAN1), LNCaP cells were transfected with lipofectamin™ 2000 in 24-well plates, and each well included 1.5 × 10^5 ^cells, 0.8 μg pGL_3_-construct, 0.4 μg pcDNA3.1-*NKX3.1*, 0.04 μg pRL-TK, 2 μl lipofectamin™ 2000 and 500 μl RPMI 1640 media without serum and antibiotics.

### Dual-luciferase reporter assays

Forty-eight hours after the transfection, the activities of Firefly luciferase in pGL_3_-constructs and Renilla luciferase in pRL-TK were determined by the dual-luciferase reporter assays following the protocol of the manufacture (Promega). The cells were rinsed with phosphate-buffered saline, and lysed with 1 × passive lysis buffer. Twenty μl of cell lysate was transferred into the luminometer tube containing 100 μl luciferase assay reagent II. Firefly luciferase activity (M1) was measured first, and then Renilla luciferase activity (M2) was determined after the addition of 100 μl Stop & Glo reagent. M1/M2 was taken as the relative luciferase activity of the pGL_3_-constructs.

### Reverse transcription-PCR

Total RNA was isolated from LNCaP and PC-3 cells using Trizol reagent (Invitrogen) 48 h after transfection with pcDNA3.1-*NKX3.1*, and expression of *PCAN1 *mRNA was determined by RT-PCR with M-MuL V reverse transcriptase (Promega) in the presence of random hexamer primers. PCR primers for *PCAN1 *were *PCAN-*F 5'-GCGATGTGCTGTGAAATCTA-3', *PCAN-*R 5'-CTTTCACATTCCCCGTGGT-3'; for *NKX3,1 *were *NKX*-F 5'-GTACCTGTCGGCCCCTGAACG-3', *NKX*-R 5'-GCTGTTATACACGGAGACCAGG-3'. A *β-actin *mRNA was amplified and used to normalize the quantity of the *PCAN1 *mRNA in RT-PCR. The primers were *β-actin*F 5'-GCTGTCAGAGTGGTTATGT-3', *β-actin*R 5'-ACATTGACGTACAGAG AGAG-3'. The PCR conditions were 94°C 3 min, 94°C 30 s, 56°C 30 s for *PCAN1*, 63°C 30 s for *NKX3.1*, 72°C 50s, 32 cycles for *PCAN1*, 26 cycles for *NKX3.1*, 72°C 6 min. The products were identified by 1.5% agarose gel electrophoresis.

### Western blot analysis of NKX3.1 protein expression

Expression of NKX3.1 in prostate cancer cells was analyzed by Western blot analysis. Briefly, total protein was extracted from PC-3 cells or LNCaP cells using lysis buffer (containing 50 mM Tris-Cl, pH 8.0, 150 mM NaCl, 0.1%SDS, 1%NP-40, 100 μg/ml PMSF) after transfection with pcDNA3.1-*NKX3.1 *for 48 h to 72 h. The protein content of the samples was measured using the BCA protein assay kit (Shenergy Biocolor Bioscience & Technology Company, Shanghai, China). Thirty μg of each protein sample was used to detect the NKX3.1 protein expression by Western blot analysis. The primary antibody was rabbit anti-human NKX3.1 (RDI, Concord MA, USA) diluted 1: 2000; the second antibody was goat anti-rabbit IgG (Sigma) diluted 1: 2000. Relative protein levels were calculated in comparison to *β-actin *as standard. Immunoblots were detected using an ECL kit (Santa Cruz, CA, USA) and visualized after exposure to X-ray film.

### Electrophoretic mobility shift assays (EMSA)

Nuclear extracts were prepared from LNCaP cells using a nuclear extraction kit (Active Motif, Carlsbad, CA, USA) following the manufacturer's instructions. Oligonucleotides corresponding to the five binding sites shown in Table 2 were synthesized as probes. Equal amounts of sense and antisense oligonucleotides of NBSs were mixed and annealed in a buffer (10 mM Tris-HCl, pH 8.0, 200 mM NaCl, 1 mM EDTA) by heating to 95°C for 5 min and cooling slowly to room temperature. The five double-stranded NBSs were labelled with digoxigenin (DIG) (Roche). Binding reactions were performed for 20 min at room temperature in a 20 μl mixture containing 0.2% (*W*/*V*) Tween-20, 1 mM EDTA, 1 mM dithiothreitol, 30 mM KCl, 20 mM HEPES (pH 7.6), 1 μg of poly (dI-dC), 0.1 μg of poly (*L*-Lys), 20 μg of nuclear extract and 0.8 ng of DIG labelled double-stranded NBS. For the competition experiment, unlabelled double-stranded NBS or mutant NBS (sequences are shown in Table 2) in 250-fold excess were added to the binding reaction mixture and incubated. For supershift assays, anti-NKX3.1 antibody was pre-incubated with the nuclear extracts at room temperature for 30 min in the binding buffer, followed by an additional incubation for 20 min at room temperature with the reaction mixtures. Bound and free oligonucleotide probes were resolved by electrophoresis on an 8% nondenaturing polyacrylamide gel in 0.25 × Tris-Boric acid (TBE) buffer. Electroblotting and chemiluminescence detection were performed based on the instructions of the manufacturer of the DIG gel shift kit (Roche, Penzberg, Germany).

### Chromatin immunoprecipitation (ChIP)

*In vivo *binding of NKX3.1 to the NBSs in the upstream region of the *PCAN1 *gene was investigated using the ChIP assay kit (Upstate Biotechnology, Inc., Lake Placid, NY, USA). Confluent human LNCaP prostate cancer cells were transfected with pcDNA3.1-*NKX3.1*. Forty-eight hours after the transfection, cells were treated with formaldehyde (1% final concentration) to cross-link NKX3.1 to the DNA. Cells were washed with cold phosphate-buffered saline and lysed in SDS lysis buffer (1% SDS, 10 mM EDTA, and 50 mM Tris-HCl pH 8.1). The lysate was sonicated to shear DNA to a length between 200 and 1000 bp. The sonicated supernatant was diluted 10-fold with ChIP dilution buffer (0.01% SDS, 1% Triton X-100, 2 mM Tris-HCl pH 8.1), and 150 mM NaCl) and incubated with anti-NKX3.1 antibody (Santa Cruz) or rabbit IgG overnight at 4°C with rotation. To collect DNA/protein complexes, salmon sperm DNA/protein A-agarose slurry was added to the mixture and incubated for 1 h at 4°C with rotation, and the DNA/protein A-agarose was pelleted by centrifugation. After extensive washing of the pellet with a series of wash buffers, the pellet was dissolved with 250 μl of elution buffer and centrifuged to remove the agarose. The supernatant was treated with 20 μl of 5 M NaCl and heated to 65°C for 4 h to reverse the NKX3.1-DNA cross-link. After treatment with EDTA and proteinase K, the supernatant was extracted with phenol/chloroform and precipitated with ethanol to recover the DNA. For PCR using the chromatin-immunoprecipitated DNA, one-tenth of the DNA was PCR-amplified using four pairs of primers (primer names and sequences are shown in Table 1) that span the five NBSs in the upstream region of the *PCAN1 *gene respectively. Twenty-five cycles of PCR at 94°C for 30 s, 60°C for 30 s, and 72°C for 30 s were performed. PCR products were analyzed in 1% agarose gels.

### RNA interference (RNAi)

pRNAT-U6.1/Neo, containing human U6 promoter, was used to generate a series of RNAi expression vectors by inserting annealed oligonucleotides between *BamH *I and *Hind *III sites. The oligonucleotides RNAi1 (5'*GATCC*CCagttcagccatcagaagtaTT CAAGAGAtacttctgatggctgaactTTTTTGGAAA3' and 3'GGGtcaagtcggtagtcttcatAA GTTCTCTatgaagactaccgacttgaAAAAACCTTT*TCGA*5'), RNAi2 (5'*GATCC*CCtcca gaacagacgctataaTTCAAGAGAttatagcgtctgttctggaTTTTTGGAAA3' and 3'GGGagg tcttgtctgcgatattAAGTTCTCTaatatcgcagacaagacctAAAAACCTTT*TCGA*5'), RNAi3 (5'*GATCC*CCtataacagctatccttactTTCAAGAGAagtaaggatagctgttataTTTTTGGAAA3'and 3'GGGatattgtcgataggaatgaAAGTTCTCTtcattcctatcgacaatatAAAAACCTTT*TC GA*5') were used for the construction of pRNAT-RNAi (1~3) targeting human NKX3.1. The oligonucleotides RiN (5'*GATC*CCCttctccgaacgtgtcacgtTTCAAGAGA acgtgacacgttcggagaaTTTTTTGGAAA3' and 3' GGGaagaggcttgcacagtgcaAAGTTCT CTtgcactgtgcaagcctcttAAAAACCTTT*TCGA*5') were used for a control vector pRNAT-RiN producing a random sequence of RNAi. pRNAT-RNAi (1–3) and pRNAT-RiN were transfected into LNCaP cells, Forty-eight hours post-transfection, the cells were harvested to detect the silencing of *NKX3.1 *with RT-PCR and Western blot. The stable cell lines transfected with pRNAT-RNAi1 or pRNAT-RiN were selected with G418 (20 μg/ml) and individual clones were isolated.

## Authors' contributions

**WL **assisted in the design of the study, carried out the cloning of expression vectors and reporter plasmid constructs, transfection studies, RT-PCR, EMSA and ChIP; helped draft the manuscript. **PZ **assisted in the design of the study, participated in the sequence alignment and use of computer database; assisted in the construction of recombinant vectors, EMSA, ChIP and RNAi; helped draft the manuscript.**WC **participated in maintenance of cell lines, performed luciferase reporter assays and assisted with the transfection experiments. **CY **carried out the isolation of RNA and purification of plasmids, assisted with the RT-PCR and ChIP. **FC **carried out Western blot and assisted with the purification of plasmids. **FK **assisted with the maintenance of cell lines and the transfection experiments. **JZ **participated in the design of the study, assisted with the revising of the manuscript. **AJ **participated in the design of the study and revision of the manuscript, helped draft the manuscript, assisted with the construction of recombinant vectors, EMSA and ChIP.

All authors have read and approved the final manuscript.
